# An Integrative Phosphoregulatory Landscape of FAK Signaling

**DOI:** 10.3390/ijms27219484

**Published:** 2026-10-23

**Authors:** Mejo George, Leona Dcunha, Mukhtar Ahmed, Althaf Mahin, Athira Perunelly Gopalakrishnan, Prashant Kumar Modi, Anoop Kumar G. Velikkakath, Rajesh Raju

**Affiliations:** 1 Center for Systems Biology and Molecular Medicine, Yenepoya Research Centre, Yenepoya (Deemed to be University), Mangalore 575018, Karnataka, India; mejomariya96@gmail.com (M.G.); prashantmodi@yenepoya.edu.in (P.K.M.); anoopkg@srinivasuniversity.edu.in (A.K.G.V.); 2 Centre for Integrative Omics Data Science, Yenepoya (Deemed to be University), Mangalore 575018, Karnataka, India; leonadcunha@gmail.com (L.D.); althafmahin99@gmail.com (A.M.);; 3 Department of Zoology, College of Science, King Saud University, P.O. Box 2455, Riyadh 11451, Saudi Arabia; mahmed1@ksu.edu.sa; 4 Central Research Laboratory, Srinivas University, Mukka 574146, Karnataka, India

**Keywords:** FAK, PTK2, cell adhesion, cell proliferation, cell migration

## Abstract

Focal adhesion kinase (FAK) is a protein tyrosine kinase that plays a major role in regulating cell adhesion, proliferation, motility, and survival. While FAK-regulatory phosphosites play significant roles in cancer progression and metastasis, the broader perspective of its phosphorylation remains largely unknown. Therefore, mapping the phosphoregulatory networks provides significant insights into targeted therapies. To characterize the phosphorylations of FAK, we systematically retrieved PubMed-indexed studies and compiled large-scale cellular mass spectrometry-based phosphoproteomics datasets. An integrative, data-driven analysis was performed on 678 qualitative and 235 quantitative cellular phosphoproteomic datasets to map the signatures of FAK phosphorylation. The study observed that the S910 modification is the most frequently detected phosphorylation outside the catalytic kinase domain. This predicts a broader regulatory role of S910 in signaling than previously understood. Further, by integrating phosphosite co-detection patterns across diverse biological contexts, we characterized FAK phosphorylations and their co-regulated Phosphosites in Other Proteins (PsOPs). The phospho-coregulatory analysis of S910 and FAK activation phosphosites Y576, Y577, and Y861 provided distinct potential phosphosite cross-talks. Furthermore, the study predicts several potential candidate substrates and interactor PsOPs, providing a framework for prioritizing phosphosites for future experimental investigation. Moreover, the study also illustrated a hypothesized model of potential FAK phosphosite-specific coregulatory associations within gastrin-associated signaling. Taken together, these large-scale observations provide a data-driven foundation for future FAK-centered experimental approaches and understanding of its multifaceted role as a potential target.

## 1. Introduction

Focal adhesion kinase (FAK), also known as protein tyrosine kinase 2 (PTK2), is a non-receptor protein tyrosine kinase encoded by the PTK2 gene [1]. The PTK2 gene is located on chromosome 8q24.3 [2], a genomic locus frequently altered across human cancers [3]. FAK plays a key role in cell adhesion, proliferation, migration, and survival through integrin and growth factor receptor-driven signaling pathways [4]. The protein has a molecular weight of approximately 125 kDa and has a length of 1052 amino acids. Structurally, FAK has three domains: the FERM domain (four-point-one-ezrin-radixin-moesin) in the N-terminus, the central kinase domain, and the FAT (focal adhesion targeting) domain in the C-terminus [5]. The N-terminal FERM domain serves as a regulatory module that interacts with growth factor receptors and c-Met, as well as downstream signaling proteins such as p53, MDM2, and Src [5]. These interactions enable the FERM domain to function as a molecular scaffold, coordinating protein–protein and protein–lipid interactions that regulate FAK activation and signal transduction. The kinase domain contains the activation phosphorylation sites Y576 and Y577. Phosphorylation of these residues maximizes FAK catalytic activity and promotes downstream signaling through pathways such as PI3K/AKT/mTOR [6]. The C-terminal region consists of the FAT domain and two proline-rich regions (PRRs). The PRRs facilitate binding to SH3 domain-containing proteins, whereas the FAT domain recruits focal adhesion proteins including paxillin, talin, vinculin, and Grb2, thereby regulating focal adhesion assembly, turnover, and intracellular signaling [5]. Additionally, intramolecular interaction between the FAT domain and the F2 subdomain of the FERM domain promotes FAK dimerization [7]. FAK functions both as a protein tyrosine kinase and as a signaling scaffold, integrating protein–protein interactions with catalytic activity [8]. In its inactive state, FAK adopts a closed, auto-inhibited conformation in which the FERM domain interacts with the kinase domain, masking the Y397 auto-phosphorylation site while still allowing FAK to function as a scaffold for protein–protein interactions [9]. FAK activation is initiated by integrin clustering at focal adhesions, where interactions between the FAT domain and adaptor proteins, including paxillin and talin, increase the concentration of FAK molecules. This enrichment promotes trans-autophosphorylation of Y397, creating a high-affinity docking site for the SH2 domain of Src family kinases, while the adjacent proline-rich (PR1) motif engages the SH3 domain of Src [10]. Subsequent Src-mediated phosphorylation of Y576 and Y577 within the kinase activation loop, together with phosphorylation of additional tyrosine residues, including Y861 and Y925, stabilizes the fully open and catalytically active FAK-Src signaling complex, thereby activating downstream pathways involved in cell migration, proliferation, survival, and mechanotransduction [11,12].

FAK has emerged as a promising therapeutic target in cancer owing to its frequent overexpression and functional contribution to tumor growth, survival, invasion, and metastasis [13]. FAK promotes tumor growth and survival, in part through activation of the PI3K/AKT/mTOR signaling axis [14], and contributes to cell-cycle progression by promoting cyclin D1 expression and modulating cyclin-dependent kinase inhibitors, including p21 and p27 [15]. FAK also supports cancer cell survival through multiple anti-apoptotic mechanisms, including PI3K/AKT/NF-κB signaling, suppression of p53-mediated apoptotic responses, and promotion of resistance to anoikis [16]. In addition, FAK regulates cell migration and invasion by coordinating integrin-dependent signaling, focal adhesion turnover, cytoskeletal remodeling, and signaling through Src, p130Cas, ERK, calpain, and Rho-family GTPases [17,18]. Importantly, these diverse biological functions are regulated by phosphorylation-dependent signaling events that can determine the functional output of FAK-associated networks. For example, FAK- and adhesion-dependent regulation of paxillin Y118 phosphorylation has been implicated in Src-induced anchorage-independent growth [19]. In colorectal cancer cells, FAK downregulation reduces AKT phosphorylation at S473 and is associated with impaired migration [20]. In cholangiocarcinoma, FAK promotes tumor development and progression through phosphorylation of YAP at Y357 [21]. Moreover, FAK directly phosphorylates GRB7 at Y188 and Y338, with these phosphorylation events contributing to integrin-dependent cell migration and proliferation [22]. Collectively, these studies underscore the importance of resolving site-specific FAK phosphorylation and substrate phosphorylation events to define context-dependent signaling networks and identify therapeutically actionable vulnerabilities.

Despite the pivotal role of FAK in cell progression, the site-specific regulatory models of its phosphorylation remain incompletely characterized and the functional significance of many individual phosphosites is still poorly understood. In particular, the extent to which distinct FAK phosphorylation sites are co-regulated with other phosphoproteins and participate in interconnected kinase signaling networks remains largely unexplored. Therefore, in this study, we applied a phosphosite co-detection-based computational approach to systematically characterize functionally relevant phosphorylation events associated with FAK using large-scale phosphoproteomic datasets. By integrating phosphosite co-detection patterns across diverse biological contexts, we characterized FAK phosphorylations and their co-regulated phosphoproteins and phosphosites. This analysis provides a systems-level understanding of the site-specific phosphorylation of FAK and establishes a framework for investigating its regulatory mechanisms, disease relevance, and potential therapeutic implications across diverse pathological conditions.

## 2. Results

### 2.1. A Curated Resource of FAK Global Human Cellular Phosphoproteomics Data

Mass spectrometry-based phosphoproteomics has advanced our understanding of cellular signaling by enabling comprehensive mapping of phosphorylation events and regulatory networks [23]. In oncology, these insights are particularly critical, as dysregulation of protein phosphorylation is a defining feature of cancer. In this regard, FAK has emerged as a central signaling hub, and its aberrant phosphorylation has been strongly associated with tumor pathogenesis [24]. Despite the identification of FAK phosphosites to date [25], comprehensive functional and regulatory insights into these phospho-modifications remain underexplored. To address this gap, we curated and compiled global human cell line phosphoproteome data from PubMed resources to systematically investigate FAK phosphorylation patterns. Screening over publicly available phosphoproteomics, we identified a total of 913 cellular datasets that reported the expression of Class-1 FAK phosphosites that were either qualitatively profiled/detected or quantitatively differential. More specifically, we identified 678 qualitative profile datasets from 119 research articles and 235 quantitative differential datasets from 56 research articles (Supplementary Tables S1 and S2). The phosphosites and proteins were mapped to UniProt accessions and gene symbols, respectively, and computational ranking within each dataset was performed to identify the most frequently detected phosphosites of FAK.

### 2.2. Identification of Frequently Detected Phospho-Modifications in FAK

**Figure 1:**
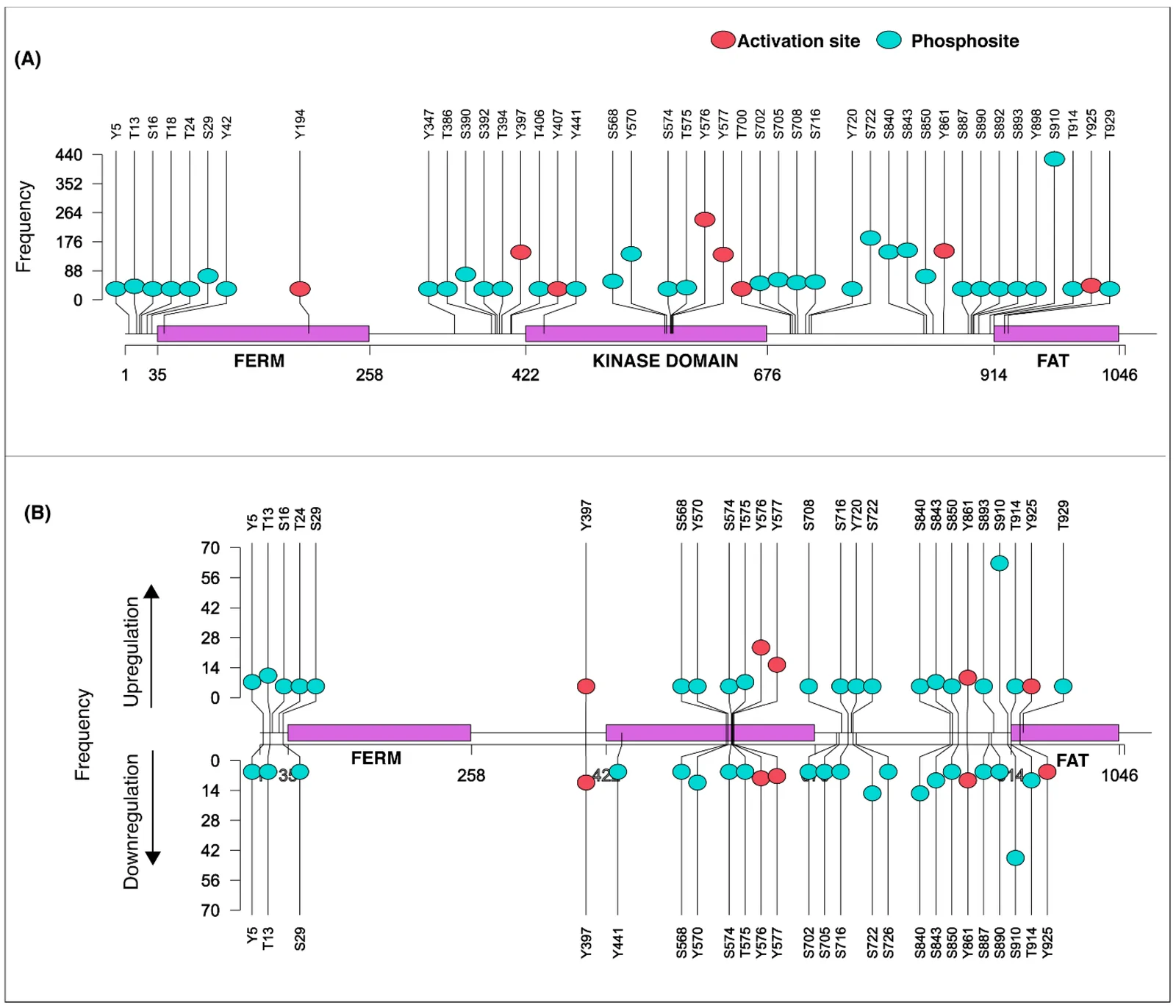
Lollipop plot representing the frequency distribution of FAK phosphorylation. (**A**) FAK phosphosites detected from profiling datasets. (**B**) FAK phosphosites detected from differential datasets. Frequency shows the number of datasets in which a phosphosite is identified/differentially regulated.

FAK is primarily characterized by its tyrosine phosphorylation [26], while serine/threonine phosphorylation remains comparatively understudied, despite evidence supporting its functional significance [27,28]. Our analysis identified serine modification S910 as a frequently detected phospho-modification in FAK with a frequency of 402 from profile data and 111 from differential data. The phosphosites Y576 and Y577 followed with respective frequencies of 295 and 168 in cellular qualitative and 35 and 26 in quantitative experimental conditions. Structurally, S910 is located in the C-terminal region adjacent to the FAT domain [29]. The FAT domain serves as a key regulatory module for FAK localization, conformational activation, and interactions with adhesion-associated signaling proteins [30]. The phosphosites Y576 and Y577 are established activation sites within the kinase domain [29]. Given the importance of FAK kinase activation in cellular proliferation and migration, the frequencies of activation sites that were among the top frequencies were also examined. This analysis showed that Y861 and Y397 have frequencies of 153 and 141 from profile data and 22 and 17 under differential experimental conditions respectively. Collectively, these annotations and frequency data designate S910 as the most frequently detected phospho-modification in FAK located outside the kinase domain (PoKD) [31]. The lollipop representation of the frequency distribution of FAK phosphosites is represented in Figure 1. This finding suggests a potential regulatory mechanism that extends beyond canonical tyrosine phosphorylation.

### 2.3. Analysis of FAK Phosphosite Co-Occurrence Patterns

**Figure 2:**
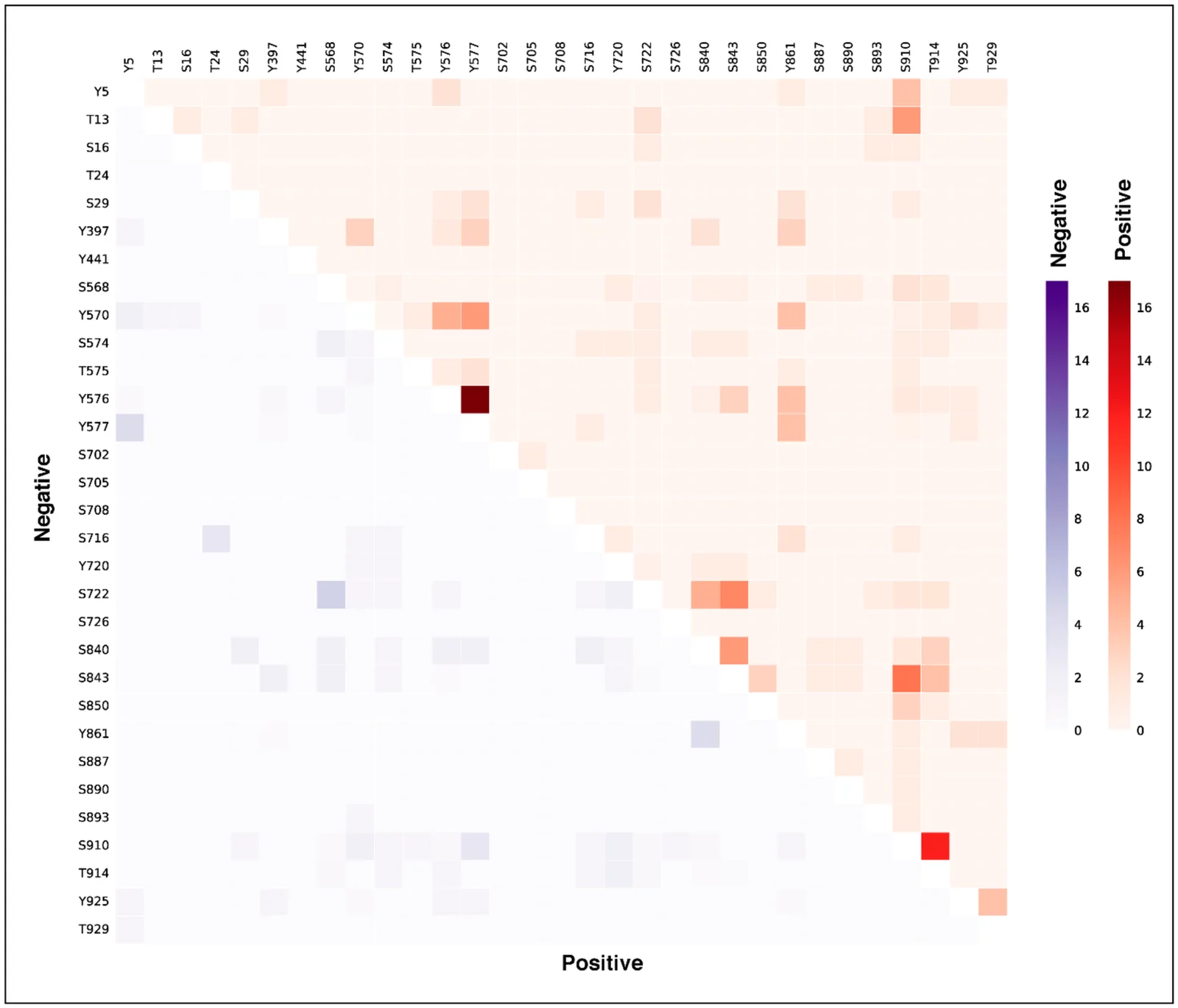
Co-occurring phosphosite pairs within FAK.

Co-occurrence of phosphosites within the same protein or across interacting proteins can reflect coordinated phosphorylation patterns and potential functional relationships within signaling pathways [32]. In this study, we examined the co-occurrence patterns of FAK phosphosites across large-scale cellular phosphoproteomic datasets to identify patterns of coordinated regulation. Differential phosphoproteomic datasets in which multiple FAK phosphosites were detected within the same experimental condition were selected for analysis. The co-occurring phosphosite pairs’ frequency was ranked according to their positive/negative co-occurrence patterns. Phosphosite pairs showing the same direction of regulation, either both upregulated or both downregulated, were classified as positive co-occurrence, whereas pairs showing opposite directions of regulation were classified as negative co-occurrence (Supplementary Table S19). For instance, Y576 was found to strongly positively co-occur with Y577 in the kinase domain, and the phosphosite S910 positively co-occurred with T914, which is present in the FAT domain, indicating coordinated phosphorylation events. However, the specific functional role of T914 remains poorly characterized. Therefore, its co-occurrence with the FAK phosphosite S910 suggests that T914 may represent a potential regulatory phosphosite. Y576 and Y577 are adjacent phosphosites located within the kinase domain of FAK, and they are associated with kinase activity. Their frequent co-occurrence is coordinated activation-loop phosphorylation and may represent an activated state of FAK that facilitates downstream signaling. The heatmap representation of co-occurring phosphosite pairs within FAK is shown in Figure 2.

### 2.4. The Co-Regulated Phosphosites in Other Proteins (PsOPs) Associated with FAK Phosphorylation

We analyzed FAK phosphorylation sites S910, Y576, Y577 and Y861 to characterize their phospho-signaling patterns using phospho-coregulation analysis. This approach identified PsOPs showing concordant or discordant phosphorylation patterns with the respective FAK phosphosites. Positive coregulation represents phosphosites showing parallel regulation, whereas negative coregulation represents phosphosites exhibiting opposite regulation patterns. Statistical significance was assessed using a FET and FDR analysis, with predefined thresholds for coregulation frequency, *p*-value, PMID support, and experimental condition-code support based on contingency tables comparing the regulation patterns of individual PsOPs with each FAK phosphosite. Using a 10% cutoff across experimental conditions, we identified 471, 28, 3, and 16 positively co-regulated PsOPs for S910, Y576, Y577 and Y861 respectively. Correspondingly, 3, 23, 12, and 3 PsOPs showed negative coregulation with these sites. The positively and negatively co-regulated PsOPs are listed in Supplementary Tables S3–S10.

**Figure 3:**
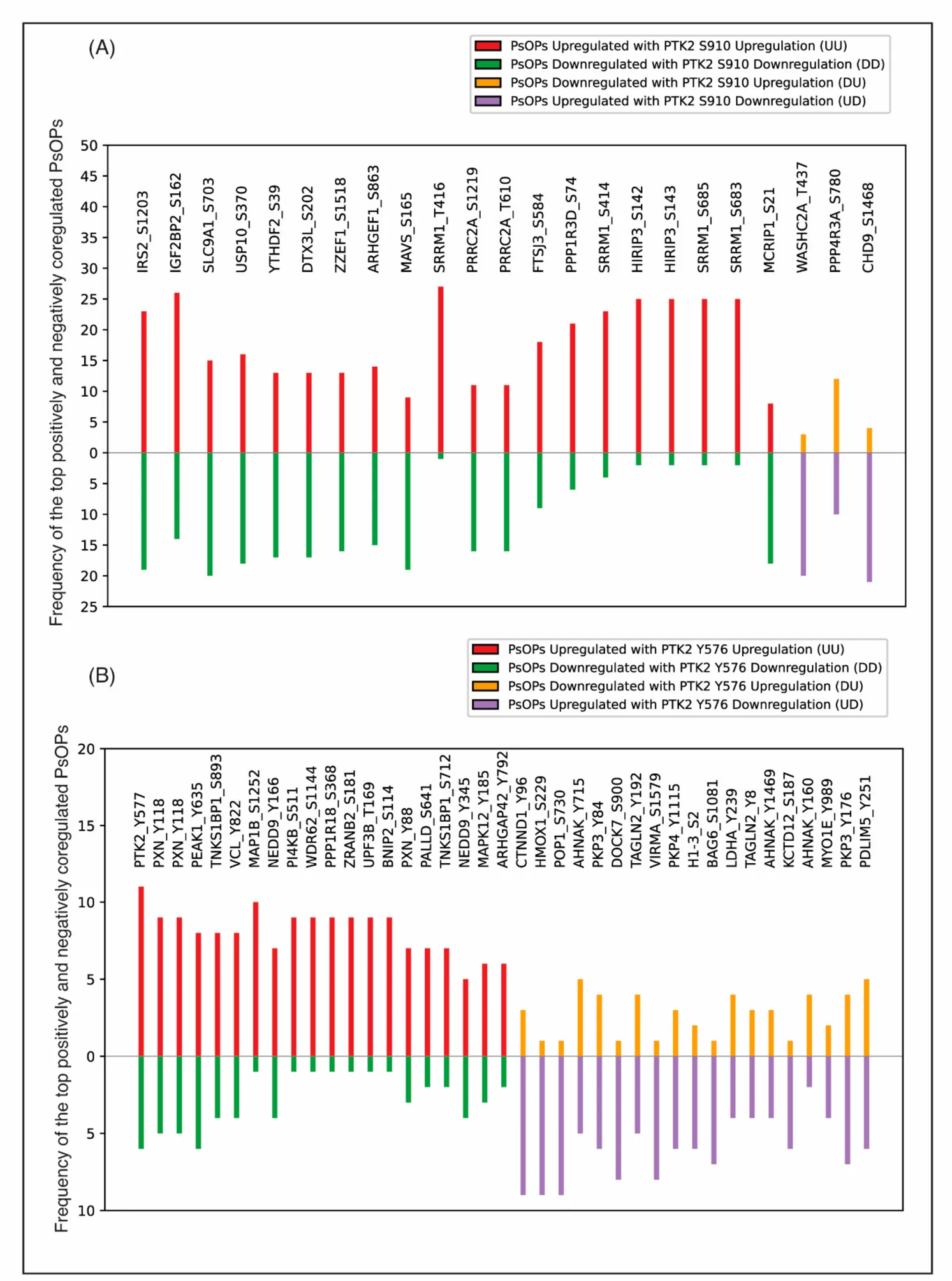
Bar plots represent the top PsOPs showing high-confidence positive (UUDD) and negative (UDDU) coregulation with FAK (**A**) S910 and (**B**) Y576 respectively.

**Figure 4:**
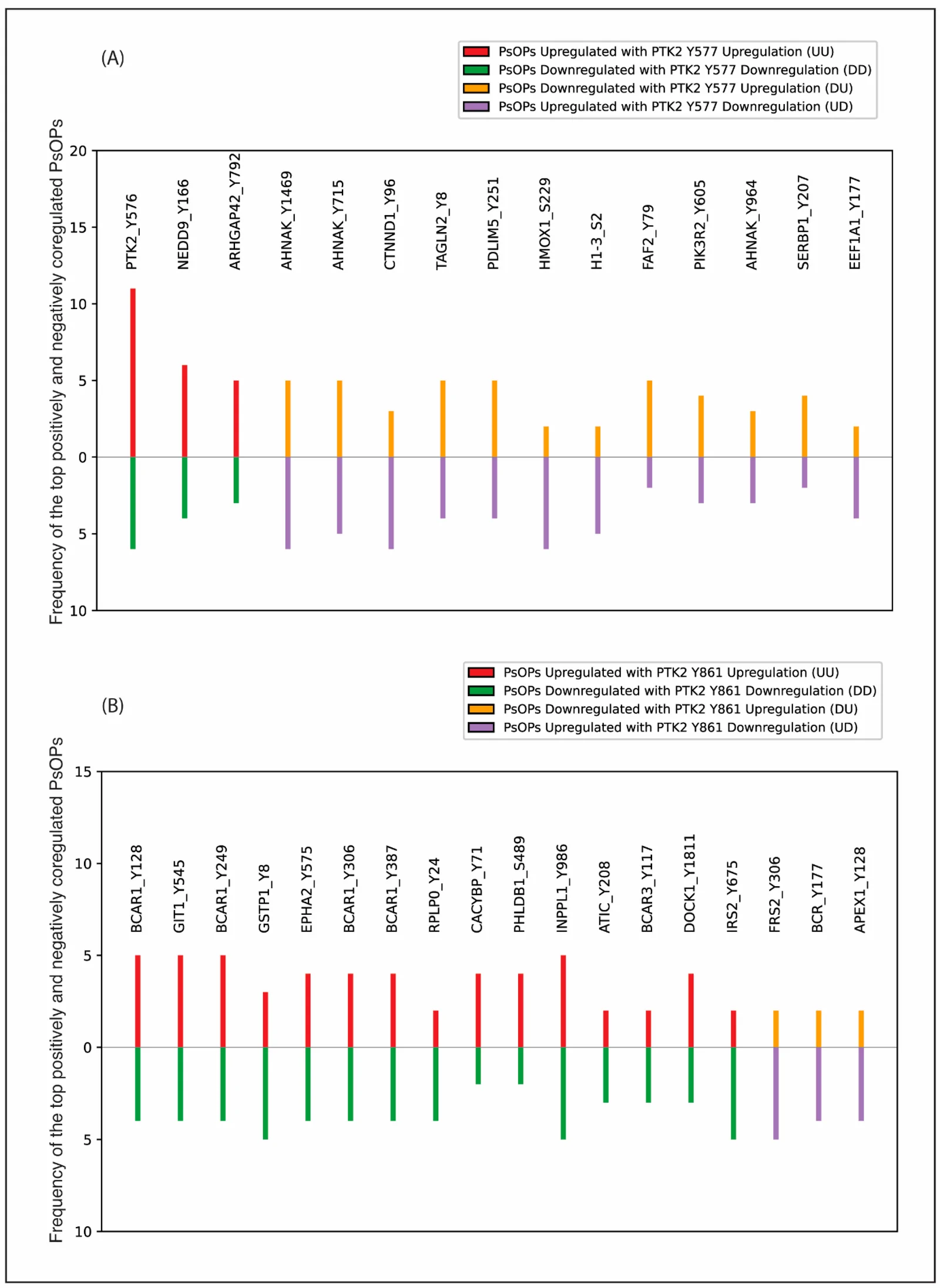
Bar plots represent the top PsOPs showing high-confidence positive (UUDD) and negative (UDDU) coregulation with (**A**) FAK Y577 and (**B**) Y861 respectively.

IRS2, IGF2BP2, SLC9A1, YTHDF2, DTX3L, ZZEF1, and MAVS were among the proteins showing strong positive coregulation with FAK S910 phosphorylation across the analyzed datasets. Among these, IGF2BP2 and YTHDF2 have previously been associated with FAK-related signaling. Lin et al. (2024) reported that IGF2BP2 expression was positively associated with FAK phosphorylation in oral squamous cell carcinoma cells [33]. Similarly, Sun et al. (2023) showed that hypoxia-induced ALKBH5 regulates ITGB1 through an m6A YTHDF2-dependent mechanism, leading to increased FAK and Src phosphorylation in epithelial ovarian cancer [34]. The PsOPs PXN (Y118), PEAK1 (Y635), VCL (Y822), and NEDD9 (Y166) showed strong positive coregulation with Y576, whereas CTNND1 (Y96), HMOX1 (S229), and POP1 (S730) showed strong negative coregulation. Similarly, NEDD9 (Y166) and ARHGAP42 (Y792) showed the strongest positive coregulation with Y577, whereas AHNAK (Y1469), AHNAK (Y715), CTNND1 (Y96), and TAGLN2 (Y8) showed the strongest negative coregulation with Y577. Previous studies have independently implicated several of these phosphosites in adhesion- and cytoskeleton-associated signaling. Sachdev et al. (2009) reported that PXN Y118 phosphorylation contributes to the regulation of Src-induced anchorage-independent growth through FAK and adhesion-dependent mechanisms [19]. Zuidema et al. (2022) reported that PEAK1 Y635 phosphorylation regulates cell migration through its association with Tensin3 and integrins [35]. Similarly, Li et al. (2025) reported that VCL Y822 phosphorylation regulates adhesion remodeling during cardiomyocyte maturation [36]. FAK Y861 showed strong positive coregulation with BCAR1 (Y128/Y249/Y306/Y387), GIT1 (Y545), GSTP1 (Y8), and EPHA2 (Y575), while showing negative coregulation with FRS2 (Y306), BCR (Y177), and APEX1 (Y128). Notably, BCAR1 tyrosine phosphorylation at Y128 and Y249 has been reported to promote carcinogenesis, cell growth, and enhanced motility [37,38]. These observations provide biological context for the identified co-regulated PsOPs and suggest their potential involvement in focal-adhesion-associated signaling networks linked to FAK activation. However, the observed coregulation does not establish direct phosphorylation or a causal relationship with FAK. The top co-regulated PsOPs associated with FAK S910, Y576, Y577 and Y861, are shown in Figure 3 and Figure 4.

### 2.5. Functional Context of PsOPs Co-Regulated with FAK S910

**Figure 5:**
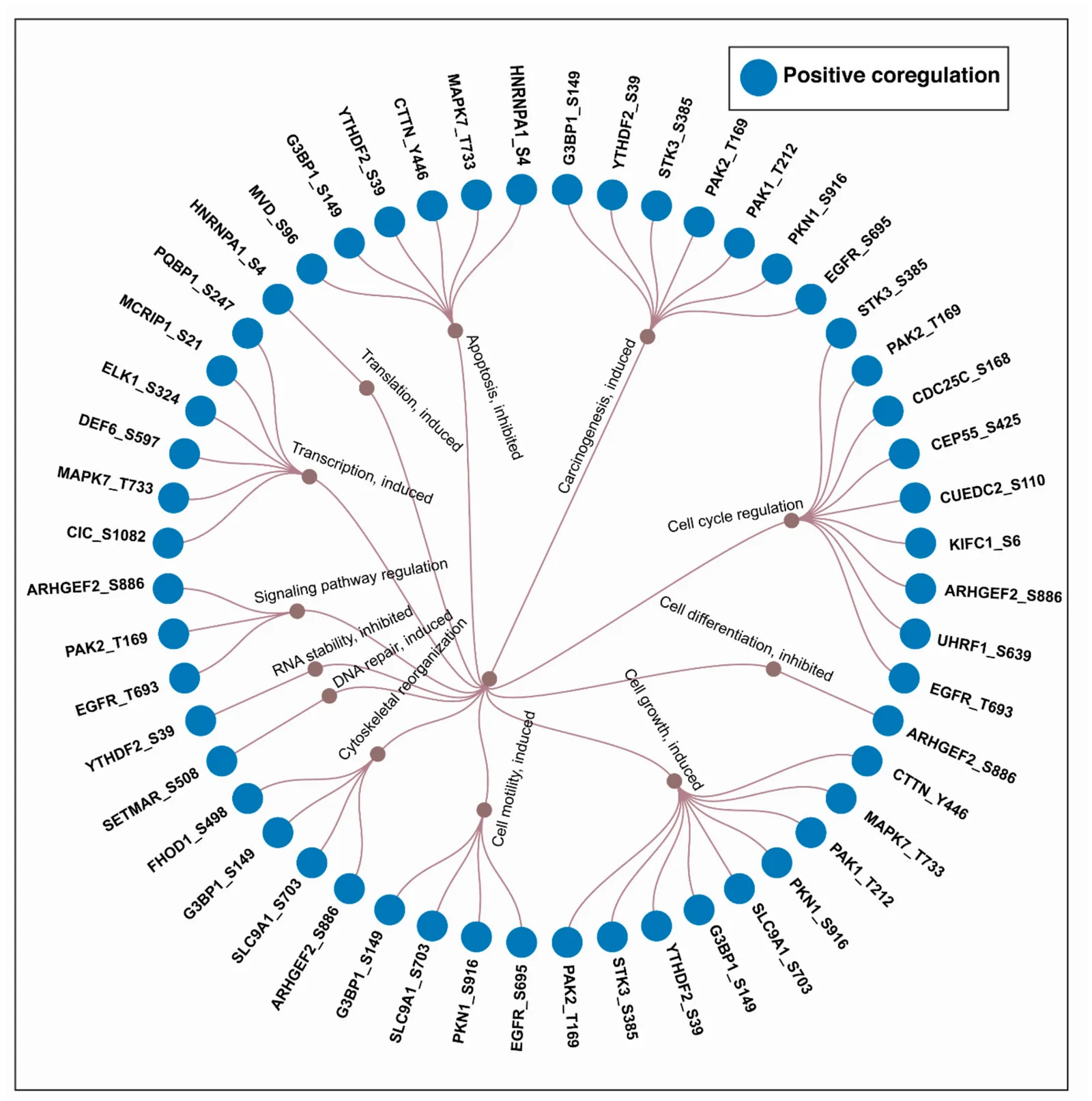
Biological processes associated with positively co-regulated (UUDD) PsOPs associated with FAK S910. Site-specific functional annotations were retrieved from the PhosphoSitePlus database.

To provide functional context for the PsOPs co-regulated with FAK S910, the identified phosphosites were annotated using the PhosphoSitePlus database. The annotated phosphosites were associated with cellular processes including cell-cycle regulation, cell growth, cytoskeletal organization, cell motility, and carcinogenesis (Supplementary Table S20). The relationships among the annotated PsOPs are presented as a dendrogram in Figure 5. As S910 is a relatively understudied FAK serine phosphosite located near the FAT domain, the identified co-regulated PsOPs provide candidates for future experimental studies to investigate their potential functional relevance.

### 2.6. Analysis of Co-Regulated Experimentally Validated and Predicted FAK Substrates

To examine potential FAK substrates, we integrated experimentally supported and computationally predicted substrate candidates from multiple complementary sources, including PhosphoSitePlus, Phospho.ELM, RegPhos 2.0, and iKiP-DB, together with the prediction framework reported by Johnson et al. (2023) [39]. The Johnson et al. study used positional scanning peptide array (PSPA) screening to define the sequence specificities of 303 human Ser/Thr kinases and generated kinase–substrate predictions based on phosphosite sequence features. Applying the reported prediction scores and a stringent 90th-percentile cutoff identified a set of putative FAK substrate candidates. As these predictions do not constitute direct experimental evidence of FAK-specific substrate relationships, they were considered as an independent computational evidence layer rather than definitive FAK substrates. These candidates were subsequently integrated with phosphoproteomic coregulation analyses to identify potential FAK-associated phosphorylation targets.

**Figure 6:**
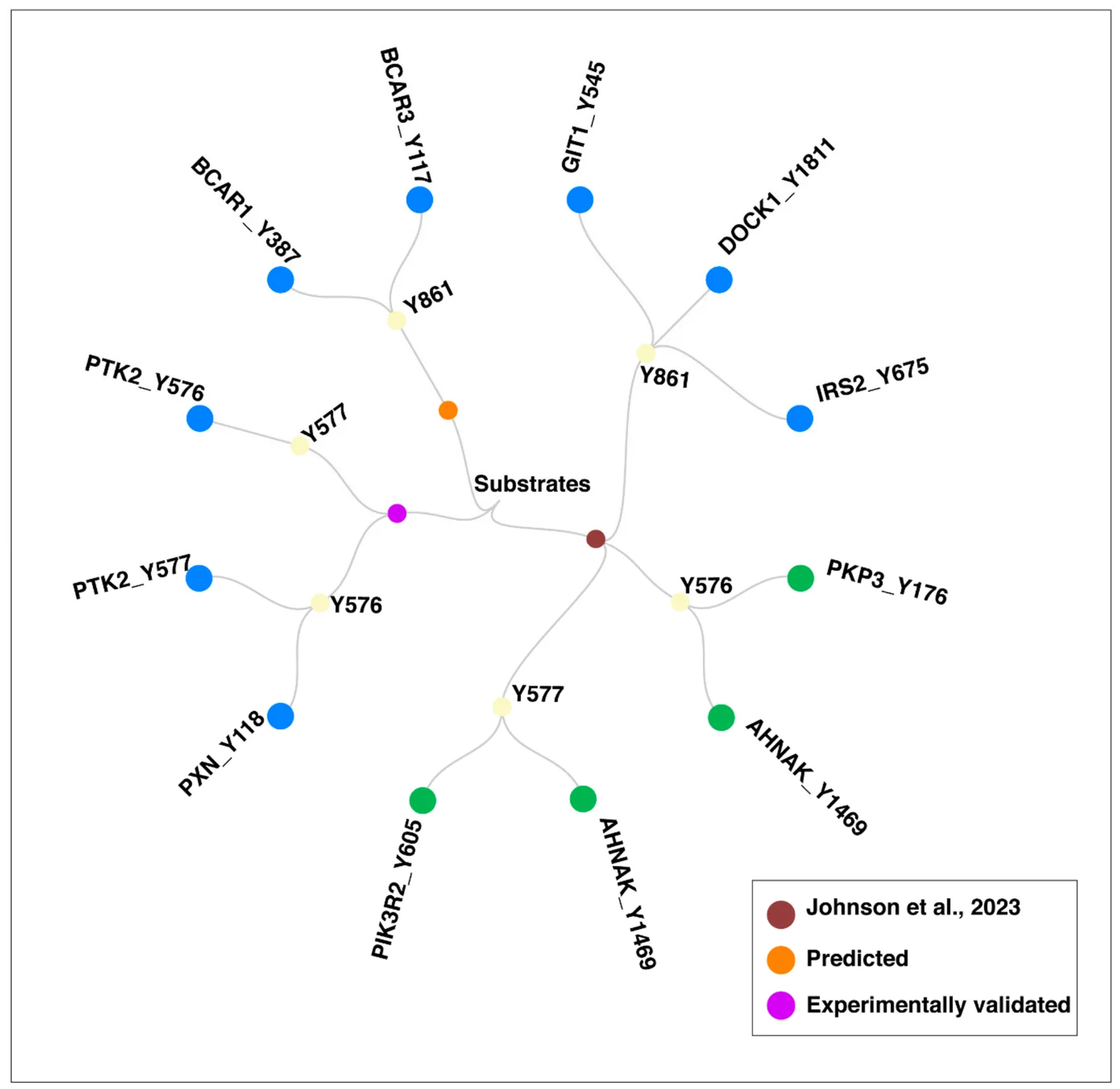
The downstream substrates of co-differentially expressed PsOPs from PhosphoSitePlus [40], Predicted sources [41,42], and Johnson et al. (2023) [39].

Using the defined FDR < 0.05 cutoff, 11 substrate candidates were identified as significantly co-regulated with the FAK phosphorylation sites Y576, Y577, and Y861 (Supplementary Table S21). Among these, PTK2 (Y577), PXN (Y118), and PTK2 (Y576) were supported by experimentally validated evidence. In addition, eight predicted substrate candidates PKP3 (Y176), AHNAK (Y1469), PIK3R2 (Y605), GIT1 (Y545), DOCK1 (Y1811), IRS2 (Y675), BCAR1 (Y38), and BCAR3 (Y117) were identified based on computational prediction. These proteins have been implicated in the positive regulation of cellular processes. Notably, PTK2, PXN, PKP3, PIK3R2, GIT1, DOCK1, and BCAR1 were identified as functionally interconnected cellular regulators, associated with cell adhesion and cytoskeletal organization (Supplementary Table S22). The identified candidate substrates and their association with the corresponding FAK phosphorylation sites are illustrated in Figure 6. These observations highlight potential substrate candidates for further experimental validation.

### 2.7. Coregulation of Phosphosites on FAK-Interacting Proteins Identifies Potential Candidates Associated with FAK Signaling

**Figure 7:**
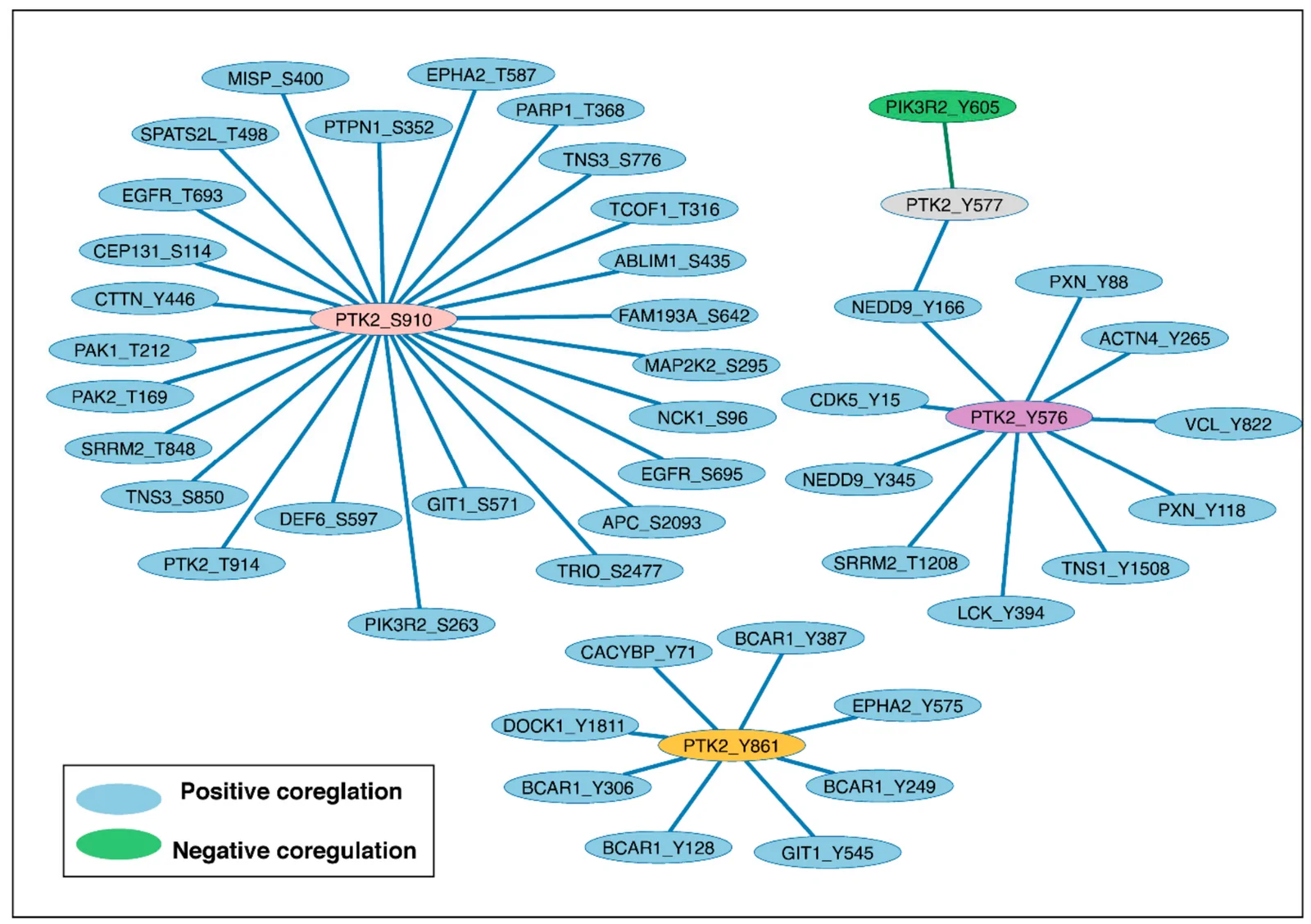
The protein–protein interactome identified across co-differentially expressed PsOPs.

To explore potential coregulation between FAK phosphorylation sites and phosphosites on its interacting proteins, we analyzed FAK binary interactors within the global phosphoproteomic datasets. This analysis identified 46 FAK-interactor PsOPs showing significant coregulation with FAK phosphosites (Figure 7). The identified proteins included PTPN1, EGFR, APC, EPHA2, CTTN, NCK1, PXN, VCL, NEDD9, ACTN4, CDK5, LCK, BCAR1, and DOCK1, among others (Supplementary Table S23). Gene ontology analysis of these FAK-interacting proteins indicated enrichment of cellular processes associated with cell adhesion and motility, consistent with the established functional involvement of FAK-associated signaling in these cellular processes.

Several of the observed PsOPs showed positive coregulation with functionally characterized FAK phosphosites. For example, LCK Y394 showed positive coregulation with FAK Y576, a phosphosite within the FAK kinase domain activation loop. LCK Y394 has previously been associated with cellular processes including cell growth, DNA repair, and cancer-related signaling. Similarly, BCAR1 Y128 and GIT1 Y545 showed positive coregulation with the FAK C-terminal phosphosite Y861. These phosphosites have previously been implicated in cellular processes involving cell adhesion, motility, cell growth, and cancer-associated signaling. Together, these observations identify phosphosites on FAK-interacting proteins that exhibit coordinated phosphorylation patterns with functionally characterized FAK sites.

We also observed several phosphosites that showed positive coregulation with the FAK C-terminal S910 phosphosite, including CTTN Y446, PAK2 T169, PAK1 T212, EGFR S695, EGFR T693, and DEF6 S597. Several of these sites have previously been reported in cancer-associated cellular contexts. For example, PAK2 T169 has been reported in the context of gastric cancer [43], while CTTN Y446 has been implicated in apoptotic signaling [44]. EGFR S695 has also been reported in relation to cellular motility and cancer-associated processes [45]. The observation of these sites within the coregulation analysis provides additional candidates for investigating potential relationships between FAK-associated phosphorylation and downstream signaling processes.

Importantly, these associations were derived from global phosphoproteomic datasets and represent coordinated phosphorylation patterns rather than direct evidence of FAK-mediated phosphorylation or kinase–substrate relationships. The analysis is therefore hypothesis-generating and is subject to limitations of heterogeneous experimental conditions. Nevertheless, the identified FAK-interactor PsOPs provide a candidate framework for prioritizing phosphosites for future experimental investigation. Selected candidates can be further evaluated using co-immunoprecipitation, site-directed mutagenesis and targeted phosphoproteomics to determine whether their phosphorylation is mechanistically associated with specific FAK phosphorylation states.

### 2.8. Potential Co-Regulated PsOPs Associated with FAK and the Gastrin Signaling Network

**Figure 8:**
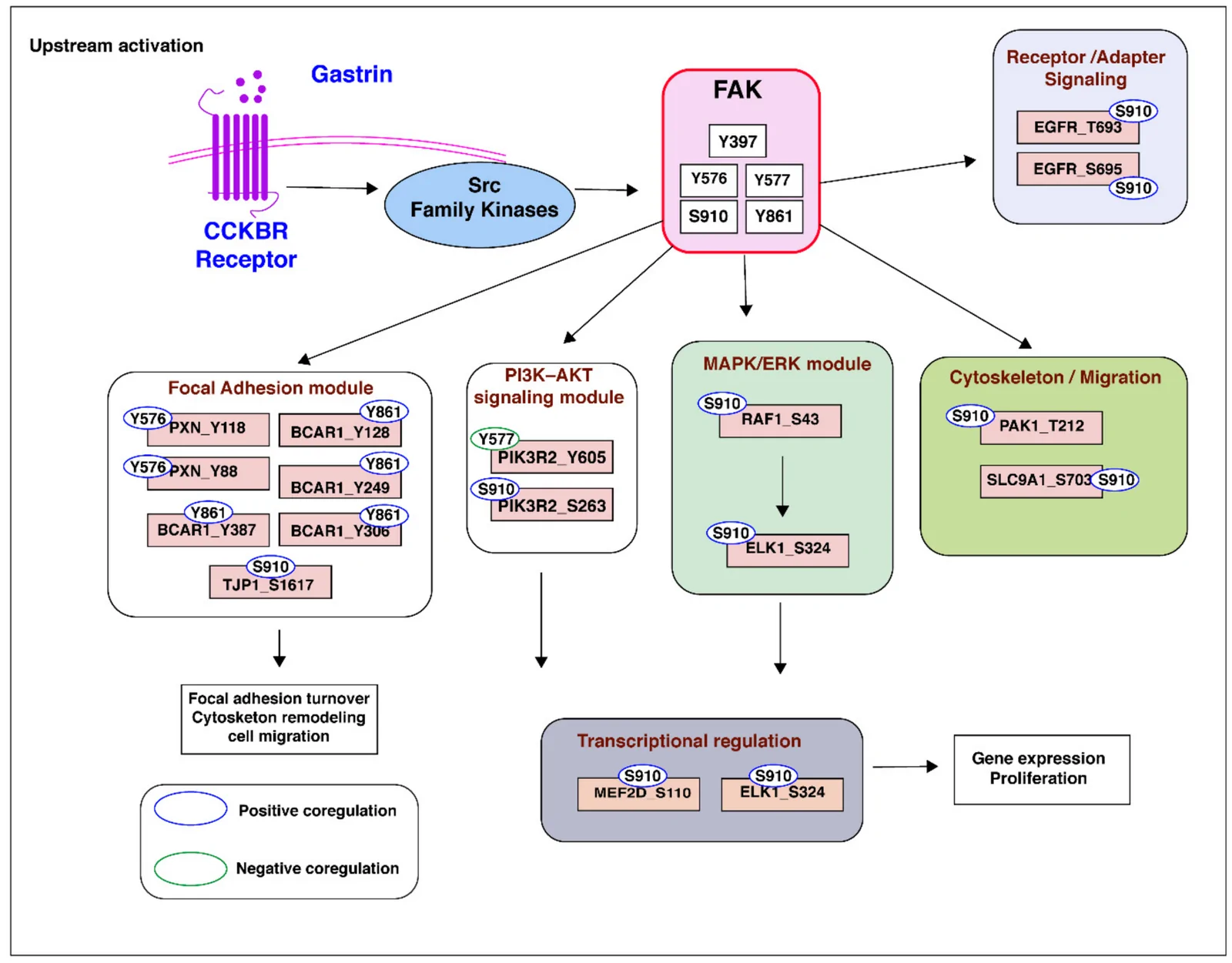
Potential FAK-associated co-regulated PsOPs within the gastrin-associated signaling network. The figure depicts positively (UUDD) and negatively (UDDU) co-regulated PsOPs associated with individual FAK phosphorylation sites, illustrating potential phosphosite-specific coregulatory associations within the gastrin-associated signaling network. The network represents a hypothesis-generating model based on phosphoproteomic coregulation patterns and previously reported gastrin FAK signaling relationships.

The co-regulated PsOPs with FDR-adjusted *p*-values < 0.05 were subjected to pathway enrichment analysis, which identified significant enrichment of the gastrin signaling pathway. The enriched proteins included PTK2, PXN, BCAR1, TJP1, SLC9A1, EGFR, PAK1, PIK3R2, RAF1, ELK1, and MEF2D. The enrichment of the gastrin signaling pathway was particularly relevant in the context of FAK signaling, as previous studies have reported FAK as a downstream signal transducer of gastrin receptor-mediated signaling [46]. Based on this reported association and our previously published gastrin signaling network [47], we mapped the identified co-regulated 19 PsOPs onto the gastrin-associated signaling network to examine their potential relationships with components involved in focal adhesion dynamics, PI3K–AKT and MAPK signaling, cytoskeletal remodeling, and transcriptional regulation. The resulting network represents a hypothesized model of potential phosphosite-specific coregulatory associations within gastrin-associated signaling. The schematic representation of the proposed phosphosite-specific coregulatory associations is presented in Figure 8.

## 3. Discussion

Protein phosphorylation plays a role in coordinating signaling networks through protein activity regulation, localization, and interactions. In cancer, aberrant phosphorylation resulting from kinase activation/downregulation rewires signaling pathways that drive tumor initiation, progression, and metastasis. The developments in MS-based phosphoproteomics have facilitated the systematic characterization of phosphorylation events, kinase–substrate networks and protein–protein interactions enabling the explanation of site-specific signaling mechanisms. In the present study, we used global phosphoproteomic data to investigate phosphosite-specific coregulatory networks associated with FAK. The use of global phosphoproteomic datasets enabled an assessment of FAK phosphorylation across diverse biological contexts, providing a broader perspective than individual studies alone. The large number of qualitative and quantitative datasets enabled the identification of FAK phosphosites that were recurrently detected across multiple experimental conditions. Systematic analysis of phosphosite detection frequencies identified residues that were repeatedly observed across the curated datasets, providing a basis for prioritizing sites for further investigation.

S910 was among the most frequently detected FAK phosphosites across the compiled phosphoproteomic datasets, indicating that this site is recurrently observed across diverse experimental conditions. However, the high detection frequency of S910 should not be interpreted as direct evidence of increased phosphorylation abundance. Nevertheless, previous experimental studies have demonstrated that S910 phosphorylation can participate in the regulation of FAK-associated signaling and adhesion dynamics. Phosphorylation at S910 has been implicated in Ras-mediated signaling, where it functions as a regulatory site rather than a canonical FAK activation site [48]. ERK phosphorylates FAK S910 and this phosphorylation generates a docking site for PIN1. The binding of PIN1 allows the phosphatase PTP-PEST to bind and dephosphorylate the Y397 activation site, thereby suppressing FAK catalytic activity and increasing focal adhesion turnover, and enhancing cancer cell migration and metastasis [48]. Thus, the frequent detection of S910 across phosphoproteomic datasets may reflect its recurrent involvement in signaling contexts associated with adhesion remodeling and cell migration, although its detection frequency alone does not establish its phosphorylation level or functional activity.

The regulatory role of S910 phosphorylation has also been identified in cardiomyocytes, where it is essential for maintaining adhesion dynamics and cytoskeletal organization. In cardiomyocytes, hypertrophic stimuli such as endothelin-1 induce FAK S910 phosphorylation through PKCδ- and Src-dependent activation. Functional analyses using the S910A mutant demonstrated that loss of S910 phosphorylation stabilizes the FAK–paxillin interaction and reduces paxillin turnover, which impairs cell spreading and sarcomere reorganization [30]. Furthermore, FAK S910 and ERK5 expression was markedly reduced in myocardial tissue from patients with dilated cardiomyopathy, suggesting that S910 phosphorylation is required for normal adhesion remodeling and maintenance of cardiac structure. Collectively, these experimental studies indicate that S910 phosphorylation can influence FAK interactions with adhesion-associated proteins and cellular adhesion dynamics beyond its canonical kinase-activation functions.

The pathway enrichment of gastrin signaling provides a potential biological context for the FAK-associated co-regulated PsOPs identified in our analysis. Gastrin, the primary hormone responsible for stimulating gastric acid secretion, has also been implicated in gastrointestinal disorders and carcinogenesis [49,50]. Its biological effects are primarily mediated through the cholecystokinin B receptor (CCKBR/CCK2R) [47]. Previous studies have reported an association between FAK and gastrin receptor-mediated signaling. Li et al. (2008) reported that FAK functions as a downstream signal transducer of gastrin receptor signaling, while Yu et al. (2006) demonstrated the involvement of FAK in CCK2R-mediated signaling [46,51]. Furthermore, a study reported that gastrin stimulates the formation of a p60Src/p125FAK signaling complex upstream of the PI3K pathway, providing experimental reports for the involvement of FAK in gastrin-associated signaling [52]. Based on this literature support and our previously published gastrin signaling network [47], we mapped the identified proteins such as SLC9A1, TJP1, EGFR, PAK1, ELK1, RAF1, PTK2, PIK3R2, MEF2D, PXN, and BCAR1 with their corresponding phosphosites onto the gastrin-associated signaling network to visualize potential FAK phosphosite-specific coregulatory relationships.

The proteins observed in the enriched pathways have been reported to be associated with gastric cancer. SLC9A1 has been reported to be upregulated in gastric cancer and to regulate cancer cell proliferation, migration, and invasion [53]. Phosphorylation of SLC9A1 at S703 has been characterized as an activity-inducing site and has been associated with the regulation of cell growth and motility [54,55]. In gastric cancer, TJP1 functions as a context-dependent molecular regulator, with its upregulation or cytoplasmic translocation reported to be associated with *Helicobacter pylori* infection and the circSMC3/miR-4720-3p regulatory axis [56,57]. EGFR has been investigated as a potential therapeutic target in gastric cancer [58,59], while PAK1 is reported to be overexpressed in gastric cancer and associated with cancer metastasis [60]. ELK1 has been identified among hypomethylated genes reported in metastatic gastric cancer, highlighting its potential relevance to gastric cancer pathophysiology and its possible utility as a biomarker [61]. RAF1 facilitates KIT signaling and has been proposed as a potential therapeutic target for gastrointestinal stromal tumors [62]. MEF2D has been reported to facilitate liver metastasis of gastric cancer cells by directly inducing H1X under IL-13 stimulation [63]. Furthermore, abnormal PXN expression has been associated with tumor progression and poor survival in patients with gastric cancer [64], while FLOT1 has been reported to promote gastric cancer progression and metastasis through BCAR1/ERK signaling [65].

Collectively, these studies provide a biological rationale for prioritizing the corresponding PsOPs for targeted knockdown/knockout experiments to determine their functional relevance to FAK-associated phosphoregulation and gastric cancer-related phenotypes, while our computational analysis provides preliminary support for their potential involvement in these regulatory networks. However, because the phosphoproteomic datasets included in the present analysis were derived from heterogeneous experimental conditions and were not specifically generated under gastrin stimulation, these associations should not be interpreted as evidence of gastrin-dependent phosphosite regulation. Instead, the observed overlap provides a hypothesis-generating framework that may indicate potential points of intersection between FAK-associated phosphoregulation and gastrin-associated signaling, which can be further investigated through gastrin-specific experimental validation.

In addition to the experimentally characterized phosphosites, many of the co-regulated PsOPs identified in this study currently lack phosphosite-specific functional annotations at the substrate, interactor, and biological process levels. Their consistent co-regulation with key FAK phosphosites suggests that they may represent previously unrecognized components of FAK-associated signaling. These uncharacterized phosphosites therefore provide potential candidates for future functional studies and experimental validation, which may help elucidate novel phosphoregulatory mechanisms and identify candidate therapeutic targets in cancer. Overall, this systems-level analysis extends the current understanding of FAK-associated signaling beyond individual phosphorylation events and provides a valuable resource for prioritizing candidate phosphosites and regulatory relationships for future investigation and experimental validation.

## 4. Methodology

### 4.1. Assembly of Global Phosphoproteome of FAK

To characterize the phosphorylation landscape of FAK, we conducted a literature search in PubMed using the search terms “phosphoproteomics” OR “phosphoproteome,” with review articles excluded. PubMed-indexed studies were manually screened to identify phosphoproteomic datasets derived exclusively from human cellular models. Studies employing human cell lines were included, whereas studies involving mouse, plant, and other species, as well as datasets derived from human tissues, were excluded. The Supplementary Files were then examined to determine the availability of phosphoproteomic datasets. Studies that did not meet the inclusion criteria or lacked relevant phosphoproteomic data were excluded from further analysis. The curated datasets represented a heterogeneous data pool containing diverse experimental conditions (cancer/non-cancer/drug treatments/infection) and different enrichment and quantification methods. Following a multi-step manual curation process, each study and its corresponding supplementary phosphoproteomic datasets were systematically examined to identify Class I FAK phosphosites, defined by a localization probability ≥ 75% or an A-score > 13. All these phosphoproteomic datasets were generated from LysC and/or trypsin-digested peptide analysis.

Further, the datasets were then classified into two categories. The first category comprised qualitative profiles, in which test and control samples were analyzed separately to compile a comprehensive list of the identified phosphopeptides. The second category consisted of quantitative differential datasets, in which phosphosite abundance in test samples was compared with that of the corresponding control samples to determine differentially regulated phosphopeptides. For differential datasets, we further defined significance as *p*-value < 0.05 and fold change values of ≥1.3 as upregulated proteins and ≤0.76 as downregulated proteins [66]. Using our in-house mapping tool, the proteins in each dataset were mapped to their respective gene symbols according to the HUGO Gene Nomenclature Committee and UniProt accession (downloaded on 5 May 2023) [67,68].

### 4.2. Identification of the Frequently Detected Phosphosites of FAK in the Phosphoproteome

Using the qualitative profile datasets, we counted the number of datasets in which each FAK phosphosite was detected. Based on the frequency of detection, phosphosites consistently detected across these datasets were considered the most frequently detected phosphosites of FAK [66].

### 4.3. Identification of Co-Regulated Phosphosites in Other Proteins (PsOPs) with FAK Phosphosites

The co-differential expression analysis was performed following the methodology previously established in our laboratory [69,70]. To explore the phosphoregulation associated with FAK, we systematically identified phosphosites from proteins showing co-differential regulation with the key FAK phosphorylation sites. These phosphosites are referred to as Phosphosites in Other Proteins (PsOPs) [70]. The positive and negative coregulations of PsOPs associated with the FAK phosphorylation sites were categorized based on their regulatory pattern with the differential datasets. All the PsOPs (o) that were upregulated (Uo) with upregulation of FAK (f) phosphosite (Uf) were categorized as UfUo and those downregulated (Do) with upregulation of FAK phosphosite (Uf) as UfDo. Similarly, DfDo and DfUo datasets were also categorized. To exclude the datasets showing contradicting coregulatory patterns, we further classified the PsOPs as positively correlated ((UfUo ∩ DfDo)–(UfDo ∪ DfUo)) termed as UfUoDfDo (UUDD) and negatively correlated ((UfDo ∩ DfUo)–(UfUo ∪ DfDo)) termed as UfDoDfUo (UDDU).

For the statistical significance of these co-differential regulation patterns among the differential datasets, a one-sided Fisher’s exact test (FET) was used and False Discovery Rate (FDR) was calculated. The differential regulation in experimental conditions was based on the 2 × 2 contingency table comprising (a) neither site detected (n_00), (b) only one site detected (n_U0 + n_D0 + n_0U + n_0D), (c) both sites detected with opposite regulation (n_UD + n_DU), and (d) both sites detected with unidirectional regulation (n_UU + n_DD) [70]. PsOPs with a differential FET *p*-value less than 0.05 and FDR *p*-value less than 0.05 were further filtered based on the ratio of Ʃ(nUfUo + nDfDo)/Ʃ(nUfDo + nDfUo) for positively correlated PsOPs and Ʃ(nUfDo + nDfUo)/Ʃ (nUfUo + nDfDo) for negatively correlated PsOPs (zero values were considered as 1 for the ratio) [70] (Supplementary Tables S3–S10).

To mitigate potential bias arising from the integration of phosphoproteomic datasets generated under diverse experimental conditions and mass spectrometry platforms, we refined the co-regulated PsOPs associated with each FAK phosphosite using the number of unique studies (PMIDs) and the number of distinct experimental conditions (experimental condition codes). We generated experimental codes to group experimental conditions involving the same stimuli under a single experimental code. The Supplementary Tables are provided with columns denoting code_UUDD; code_count_UUDD and code_UDDU; code_count_UDDU. The experimental code confidence value for positive coregulation was calculated by subtracting the negative coregulation experimental code count from the positive coregulation code count, and vice versa for negative coregulation (column denoting UUDD_Code_confidence and UDDU_Code_confidence). Similarly, we determined the number of unique PMIDs contributing to the phosphoproteomic data and calculated a PMID confidence value for both positive and negative coregulation (column denoting pmid_UUDD, pmid_count_UUDD, pmid_UDDU, pmid_count_UDDU, UUDD_PMID_confidence and UUDD_PMID_confidence). We then applied the experimental code confidence and PMID confidence cutoffs of ≥3 to these positive and negative coregulations. Specifically, PsOPs showing either positive or negative coregulation were considered high-confidence only when they satisfied the following criteria: a differential Fisher’s exact test (FET) *p*-value less than 0.05, FDR *p*-value less than 0.05, a ratio of at least 10% between Σ (nUcU + nDcD) and Σ (nUcDo + nDcUo), and support from at least three unique studies and three distinct experimental conditions (Supplementary Tables S3–S10). To address the potential non-independence of experimental conditions originating from the same publication, we provide the number of supporting PMIDs and the corresponding experimental condition counts for each FAK PsOP association in Supplementary Tables S11–S18. This information provides transparency regarding the distribution of experimental evidence across publications. The PMID and experimental condition-based confidence measures were employed to characterize the distribution and reproducibility of supporting evidence across independent publications and distinct experimental conditions, while reducing the potential influence of repeated experimental conditions. However, these measures do not constitute a formal statistical adjustment accounting for the non-independence of multiple experimental conditions derived from the same study. Accordingly, the resulting confidence measures should be interpreted as descriptive indicators of study-level and experimental condition support, rather than as a formal statistical correction for dataset non-independence.

### 4.4. Analysis of Co-Regulated FAK-Associated PsOPs

**Figure 9:**
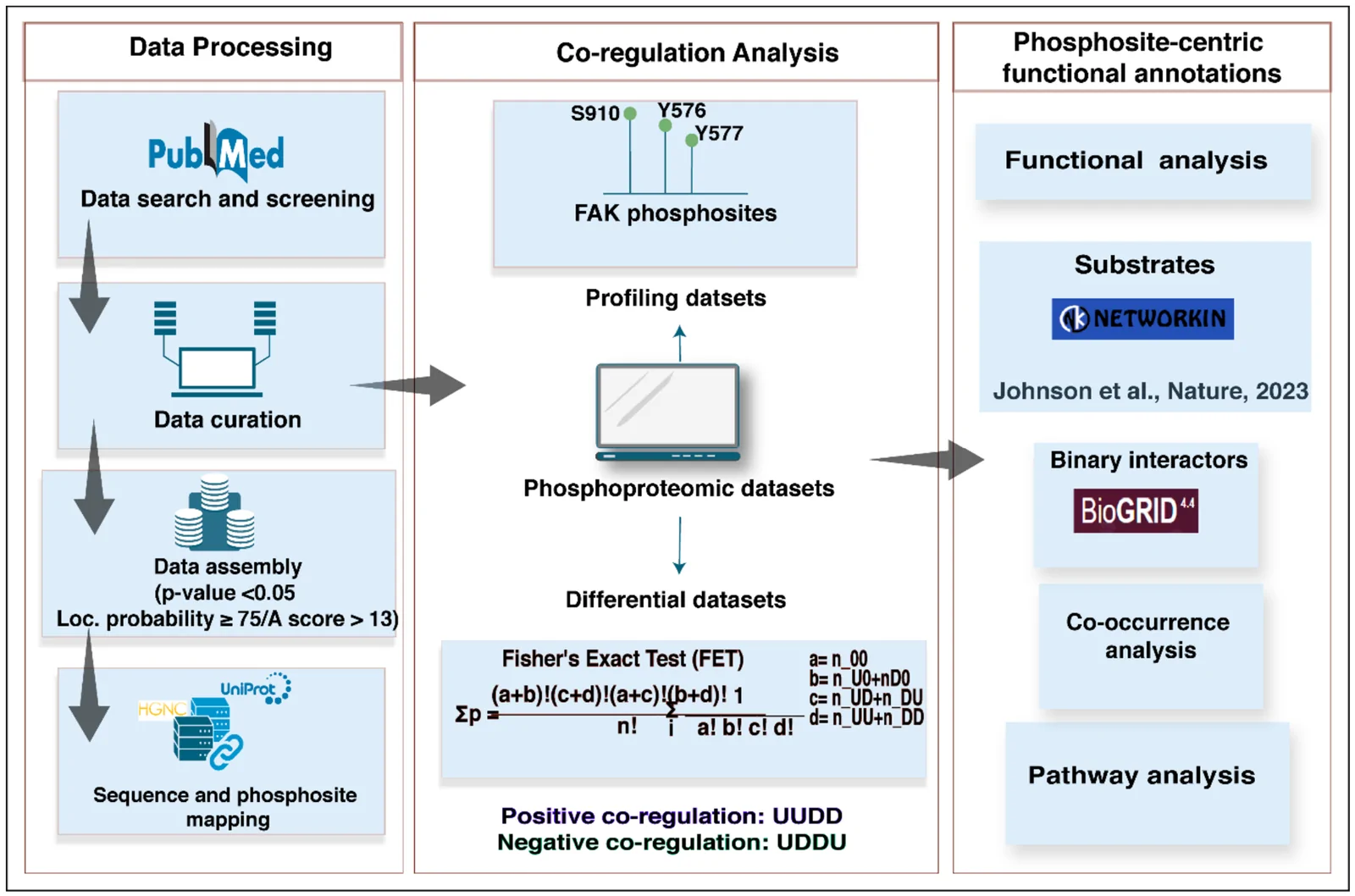
The workflow used for the data analysis of global phosphoproteomics data [39].

The high-confidence PsOPs were used for subsequent analysisof (a) experimentally known downstream substrates of FAK listed in PhosphositePlus [71]; (b) predicted kinases and substrates based on multiple prediction tools such as NetworKIN and Automatic Kinase-specific Interactions Detection (AKID) [40], In Vitro Kinase-to-Phosphosite Database (iKiP-DB) [41] and also kinases/substrates of FAK derived from the study by Johnson et al. (2023) [39]; (c) the experimentally known protein–protein interactors inclusive of the substrates extracted from databases such as the Human Protein Reference Database (HPRD) [72], Biomolecular Interaction Network Database (BIND) [72], BioGRID [73], ConsensusPathDb [74], CORUM comprehensive resource of mammalian protein complexes [75], and RegPhos [42]. The workflow is represented in Figure 9.

## 5. Limitations of the Study

Our study provides a systems-level view of FAK phosphorylation through the integration of large-scale mass spectrometry-based phosphoproteomic datasets. However, several limitations should be considered. First, the use of publicly available datasets may introduce publication and dataset-selection bias, as the available literature may not represent the full spectrum of FAK phosphorylation states or experimental conditions. In addition, the curated datasets were generated using diverse mass spectrometry platforms, phosphopeptide enrichment strategies, sample types, experimental conditions, and data-processing workflows. Differences in phosphopeptide enrichment efficiency, mass spectrometry sensitivity, peptide ionization, and site localization may influence phosphosite detectability and consequently the observed frequency of individual phosphorylation sites. Therefore, the high detection frequency of a phosphosite, including FAK S910, should not be interpreted as direct evidence of its phosphorylation abundance, occupancy, or functional importance. Because of the large number of curated datasets, limited availability of raw data, and absence of a common reprocessing pipeline, unified normalization and batch-effect correction across all datasets were not feasible. To reduce the potential influence of dataset heterogeneity, we applied multiple confidence criteria, including FET, FDR, experimental condition confidence, and PMID-based confidence. Co-regulation was assessed using a 2 × 2 contingency framework based on the patterns of site detection and directional regulation, followed by FET and FDR filtering and correlation-ratio criteria. The PMID and experimental confidence measures were used to characterize the distribution supporting evidence across publications and distinct experimental conditions and to reduce the potential influence of repeated experimental conditions. Despite these measures, residual non-independence and dataset-specific biases cannot be completely excluded. However, these measures do not constitute a formal statistical adjustment for within-publication dependence. Thus, the resulting confidence measures should be interpreted as descriptive indicators of study-level and experimental condition support rather than as a formal correction for dataset non-independence.

Finally, co-regulation represents a statistical association and does not establish a causal relationship between FAK phosphorylation and the regulation of the corresponding PsOPs. Similarly, the co-occurrence or co-regulation of phosphosites does not establish direct kinase–substrate relationships or functional dependence. Therefore, the identified high-confidence PsOPs should be considered as candidate components of FAK-associated phosphoregulatory networks rather than experimentally established causal regulators. Functional validation using approaches such as targeted knockdown/knockout, phosphosite-specific mutagenesis and in vitro kinase assays will be required to determine their mechanistic roles. Collectively, these considerations highlight the hypothesis-generating nature of the present analysis while providing a framework for prioritizing phosphosite-specific relationships for future experimental investigation.

## 6. Conclusions

This study provides a phosphosite-centric view of FAK-associated signaling through the integration of large-scale qualitative and quantitative phosphoproteomic datasets, enabling systematic characterization of FAK phosphorylation and its associated co-regulatory networks. FAK S910 was frequently detected across the curated phosphoproteomic datasets and diverse experimental contexts, indicating that this site was recurrently observed and supporting its consideration for further investigation of its potential regulatory role. Phosphosite-specific co-regulation analyses of S910, Y576, Y577, and Y861 identified co-regulated phosphorylation networks associated with cell adhesion, migration, cytoskeletal remodeling, cell-cycle regulation, intracellular localization, and cancer-related signaling processes. Integration of experimentally validated and predicted substrates, together with protein–protein interaction and phosphosite annotations, further supported the biological context of these networks and identified numerous previously uncharacterized PsOPs as candidate components of FAK-associated signaling. The observed overlap of FAK-associated co-regulated PsOPs with the gastrin signaling network further provides a hypothesis-generating framework for investigating potential intersections between FAK-associated phosphoregulation and gastrin-associated signaling in gastrointestinal cancer and related disorders. Overall, this study provides a systems-level framework for investigating phosphosite-specific FAK signaling and a resource for prioritizing candidate phosphosites for mechanistic studies, substrate validation, and functional investigation. More broadly, the findings demonstrate the utility of phosphosite-centered network analysis for identifying previously unrecognized candidate signaling relationships that may be overlooked by conventional protein-level analyses, thereby contributing to a more detailed understanding of kinase-associated phosphoregulation in cancer.

## Data Availability

The original contributions presented in this study are included in the article/Supplementary Material. Further inquiries can be directed to the corresponding author.

## Supplementary Materials

The following supporting information can be downloaded at: https://www.mdpi.com/article/10.3390/ijms27219484/s1.
